# Put some pep in your step: engineering C_4_ photosynthesis into rice using maize PEPC

**DOI:** 10.1093/plphys/kiag388

**Published:** 2026-06-17

**Authors:** Alison R Gill

**Affiliations:** Assistant Features Editor, Plant Physiology, American Society of Plant Biologists; Australian Research Council Centre of Excellence in Plants for Space, Adelaide University, Urrbrae, Australia

Plants use one of three photosynthetic pathways, with the most common being C_3_ photosynthesis where carbon dioxide (CO_2_) is directly fixed by ribulose bisphosphate carboxylase/oxygenase (Rubisco). Rubisco is inefficient, however, as it can also fix oxygen, resulting in energetically expensive photorespiration (Cackett et al 2025). To overcome this, some plants evolved a more efficient C_4_ pathway that concentrates CO_2_ around Rubisco and minimizes photorespiration (Sage 2004). In C_4_ photosynthesis, phosphoenolpyruvate carboxylase (PEPC) first fixes CO_2_ into oxaloacetate, which is subsequently converted to malate or aspartate. These metabolites act as carbon shuttles from the mesophyll to the bundle sheath cells (Fig. 1a). Malate also functions as a feedback inhibitor of PEPC, with its inhibitory effect dependent on the enzyme's phosphorylation state (phosphorylated PEPC is less sensitive to feedback inhibition), which is regulated by light. Carbon concentrating mechanisms (CCMs) in C_4_ plants confer greater photosynthetic efficiency and improve nitrogen-, water-, and light-use efficiencies (Sage 2004).

**Figure 1:**
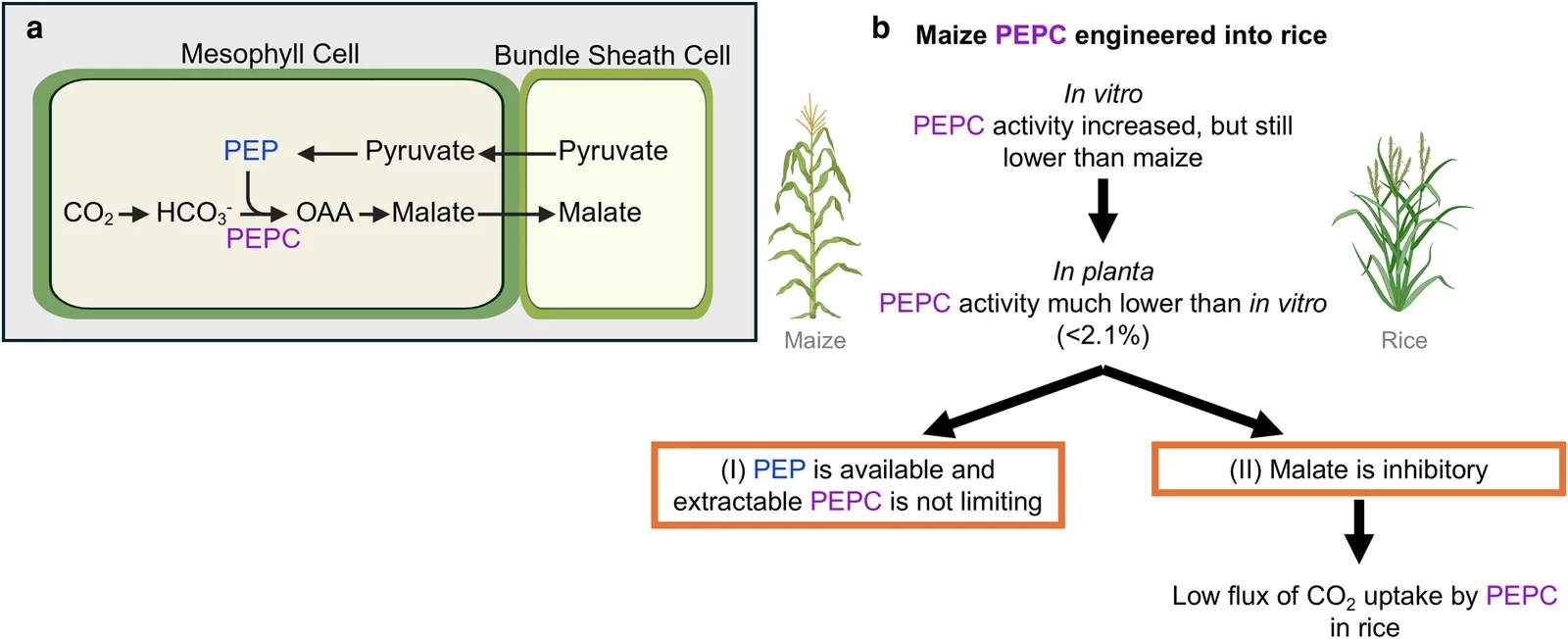
a) Simplified diagram of C_4_ photosynthesis, focused on the PEPC pathway across the mesophyll and bundle sheath cells. b) Process of elucidating why there is a low flux of CO_2_ uptake by PEPC in engineered rice with maize PEPC. The orange boxes highlight the key findings of Om et al. (2026). PEP is highlighted in blue and PEPC in purple. Created in BioRender.

Rice, a staple crop for much of the world, undergoes C_3_ photosynthesis, which constrains photosynthetic efficiency and yield, particularly under high temperatures or low nutrient availabilities. To prevent mass global malnutrition, rice yields must increase by 50% over the next 20 years. Increases of this magnitude are only possible by improving photosynthetic efficiency (Hibberd et al 2008; Cackett et al 2025), so there have been concerted efforts over the past two decades to introduce the C_4_ photosynthetic pathway into rice (von Caemmerer et al 2012; Yoon et al 2020; Ermakova et al 2021; Cackett et al 2025). For instance, this has been the focus of the C_4_ Rice Project, a large international consortium working to improve photosynthetic efficiency and enhance yields in rice by engineering both photosynthetic metabolism and anatomy (https://c4rice.com).

A major metabolic limitation in engineering C_4_ photosynthesis into rice is the subsequent low flux of CO_2_ uptake catalyzed by PEPC. This was thought to be due to reduced PEPC accumulation or low PEPC activity owing to limited availability of PEP, the enzyme's substrate (Ermakova et al 2021). While in vitro assays (i.e., using ideal conditions outside the target plant) of PEPC activity are promising, when *in planta*, the carbon flux remains limited. To successfully engineer C_4_ rice, it is necessary to understand the limitations of PEPC-dependent CO_2_ capture.

In a recent article published in *Plant Physiology*, Om et al (2026) generated two rice lines expressing either genomic or coding sequences of the C_4_ isoform of maize PEPC (hereafter referred to collectively as PEPC-OE lines) to determine the biochemical functioning of C_4_ PEPC in rice. To test in vitro PEPC activity, the authors measured PEPC activity from tissue extracts and visualized nonphosphorylated and phosphorylated PEPC in leaves via immunolocalization. ^13^CO_2_ labeling, metabolite extractions, and liquid chromatography–tandem mass spectrometry (LC-MS/MS) were used to measure *in planta* PEPC activity. Om et al (2026) also measured PEPC's kinetic properties and allosteric regulation (i.e., the modulation of protein activity) by malate and glucose 6-phosphate, both key components involved in CO_2_ fixation.

Om et al (2026) investigated whether extractable PEPC capacity and the availability of the enzyme's substrate, PEP, were limiting in transgenic rice. Both PEPC-OE lines showed higher PEPC activity than wild-type (WT) rice, with genomic DNA lines having greater activity than coding DNA lines, potentially due to higher *PEPC* gene expression and the presence of introns, which may have conferred higher transcript stability. While PEPC content in the genomic DNA lines was sufficient to drive the CCM, the PEPC activity was still much lower than in maize and was even lower once tested *in planta*; *in planta* PEPC activity was less than 2.1% of in vitro PEPC activity. The authors concluded that PEP availability, determined by measuring PEP concentration in the cytosol using LC-MS/MS, and PEPC capacity were not limiting (Fig. 1b(I)).

So, if PEPC and PEP were not limiting, why was CO_2_ uptake via PEPC still low in the engineered rice? Om et al (2026) attributed this to malate inhibition (Fig. 1b(II)), compounded by a mismatch in light-dependent activation; PEPC in rice was phosphorylated in the dark, whereas PEPC in maize was phosphorylated in the light. As phosphorylated PEPC is less sensitive to malate feedback inhibition, this keeps PEPC active at high malate concentrations in maize, highlighting the importance of light activation. In contrast, PEPC in rice is mostly in the nonphosphorylated state and is therefore highly sensitive to malate, resulting in strong allosteric inhibition that limits the initiation of the CCM. To effectively regulate PEPC in rice, it is likely that light-dependent phosphorylation or other posttranslational modifications are required.

Om et al (2026) navigate the bottleneck of the low flux of CO_2_ uptake by PEPC in rice by showing that the CCM was limited by malate feedback inhibition and low phosphorylation of PEPC in the light and identifying other posttranslational modifications that may be occurring in maize. To successfully engineer C_4_ photosynthesis into rice and drive PEPC's activation in the light, future studies must remove these constraints. Other bottlenecks remain, including optimizing subsequent steps of the C_4_ pathway, improving cell anatomy to increase bundle sheath chloroplast content and vein formation, and achieving appropriate Rubisco abundance and localization. Together, these advances will be central to improving photosynthetic efficiency and yield in rice by introducing the C_4_ pathway, with meaningful importance for food security globally.

**Recent related articles in *Plant Physiology*:**

- Cackett et al (2025) investigated the potential to increase chloroplast compartment size in rice bundle sheath cells, which could be used to engineer rice leaves to behave like C_4_ plants and thereby improve their photosynthetic efficiency.
- Sales et al (2025) studied the efficiency of C_4_ photosynthesis in maize under fluctuating light and suboptimal temperatures. They found that maize can buffer light transitions at room temperature to maintain energetic efficiency, adding to the body of knowledge about high CO_2_ assimilation in C_4_ plants.
- Fan et al (2024) compared photorespiration in C_3_ and C_4_ grasses, including rice, and discovered that leaf dark respiration is time-dependent.

## Data Availability

No new data were generated or analyzed for this News and Views article.
